# Editorial: Adaptations and responses to respiratory interventions

**DOI:** 10.3389/fphys.2026.1900228

**Published:** 2026-06-25

**Authors:** Daniel H. Craighead, Sophie Lalande

**Affiliations:** 1 School of Kinesiology, University of Minnesota, Minneapolis, MN, United States; 2 Department of Integrative Physiology, University of Colorado Boulder, Boulder, CO, United States

**Keywords:** apnea, breathing, hypoxia, inspiratory muscle training, pulmonary function

Respiratory interventions, such as Yogic breathing, resisted efforts, intermittent hypoxia, and voluntary hyperpnea, are popular and generally accessible lifestyle strategies that are purported to improve physiological function and decrease disease risk (Illidi et al., 2023). Currently, there is considerable popular interest in breathwork and other similar topics, though there remain significant gaps in our understanding as to the intermediate and long-term health benefits of the various respiratory-related interventions and their potential underlying mechanism(s). The Research Topic “*Adaptations and Responses to Respiratory Interventions*” was designed to gain clarity surrounding the benefits and mechanisms underlying such interventions. This Research Topic brought together original research, reviews, and perspectives on a broad range of issues related to respiratory interventions. Together, these articles demonstrate the breadth of this area of research and highlight areas for continued inquiry.

Acute intermittent hypoxia, a respiratory therapy in which brief periods of hypoxia alternate with brief periods of normoxia, produces numerous physiological effects, including increased erythropoietin (EPO) production (Wojan et al., 2021). Harding et al. evaluated the effects of four consecutive days of acute intermittent hypoxia, consisting of fifteen 90-second bouts of breathing 9% O_2_, on hemoglobin mass in young healthy adults. The authors found that neither EPO nor hemoglobin mass increased with this intervention, likely because the brief hypoxic intervals and short overall duration of the acute intermittent hypoxia protocol were insufficient to stimulate EPO production.

Apnea is defined as periods where breathing is halted. Andersson et al. examined the impact of apnea, alone and in combination with cold-water face immersion, on the time course of pulmonary gas exchange. The authors also examined how pulmonary gas exchange aligned with the well characterized cardiovascular responses to apnea. The main findings were that 1) pulmonary O_2_ uptake decreases during the first 30–45 seconds of apnea down to a level below eupnic baseline; 2) this time course for pulmonary O_2_ uptake aligned with reductions in heart rate and cardiac output during apnea; and 3) that apnea combined with cold-water face immersion, which stimulates the diving reflex, caused greater reductions in pulmonary O_2_ uptake than apnea alone. This study confirmed that the pulmonary oxygen store is relatively preserved during apnea.

Multiple reviews assessed the effectiveness of exercise interventions of pulmonary function in patient populations. In a narrative review, Guo et al. evaluated the effects of exercise interventions on pulmonary function in patients with respiratory conditions from the acute phase of disease through the long-term sequalae. The review comprehensively covers how aerobic exercise, resistance exercise, and more respiratory-focused exercise such as inspiratory muscle training and Tai Chi, can be incorporated with standard therapies across the acute-to-chronic pulmonary rehabilitation spectrum. Mechanisms of action also are discussed. A meta-analysis by Li et al. examined the impact of adherence to American College of Sports Medicine (ACSM) exercise guidelines (Garber et al., 2011) on pulmonary function and quality of life in patients with asthma. The 18 studies included in this meta-analysis were broad; while most investigated aerobic exercise interventions, some focused on yoga, pulmonary rehabilitation, or inspiratory muscle training. Half of the studies were considered adherent to the ACSM guidelines, while the other half of studies were not. In general, it was found that exercise interventions improved pulmonary function in asthma patients, regardless of adherence to ACSM guidelines; however, quality of life was improved only with highly adherent interventions. This meta-analysis speaks to the benefits that broad and varied types of physical activity can have for patients with asthma.

Respiratory muscle training, where resistance is applied to inspiration and/or expiration to strengthen the respiratory muscles, was examined in three publications in this Research Topic. Kang et al. looked to see if respiratory muscle training paired with mobile app-based biofeedback was superior to respiratory muscle training alone for improving pulmonary function in healthy young adults. After four weeks of training, forced expiratory volume in 1 second (FEV1) was improved similarly in both groups. FEV1/forced vital capacity also was improved in both groups, but to a greater extent in the app-based biofeedback condition. There also was a curious increase in handgrip strength in the biofeedback group, though no between-group differences for this finding were observed. In a perspectives article, Lapierre-Nguyen et al. discussed the potential for high-resistance inspiratory muscle strength training, a time-efficient form of respiratory training (Vranish and Bailey, 2015), for improving health in patients with chronic kidney disease. Patients with chronic kidney disease are more likely to die from cardiovascular disease than to progress to end-stage kidney disease (Sarnak et al., 2003); therefore, interventions that improve cardiovascular health in patients with chronic kidney disease are necessary. The authors posit that high-resistance inspiratory muscle strength training may be a promising intervention in this patient group. Furthermore, the time-efficient nature of this intervention which can be performed at home may boost adherence in chronic kidney disease patients, who generally struggle to adhere to exercise programs. Finally, Ibanez-Pegenaute et al. reported the results from a quasi-experimental trial of inspiratory muscle training in instrumentalist musicians. The authors looked at the effects of 12 weeks of twice daily high-intensity, low-repetition inspiratory muscle training *vs*. control on maximal inspiratory and expiratory pressures. Both maximal inspiratory and expiratory pressure increased in the inspiratory muscle training group relative to control. The authors also observed that about two thirds of participants would be considered responders to inspiratory muscle training, defined as an increase in maximal inspiratory and expiratory pressure greater than the minimal clinically important difference of 25 cmH_2_O.

This Research Topic attracted broad submissions about respiratory interventions and health. Common themes that emerged were apnea/hypoxia, inspiratory muscle training, and the efficacy of more conventional exercise training for improving pulmonary function. The manuscripts collected here answered some important research questions but also raised many future points for inquiry. We hope the broad articles collected here will stimulate further research on respiratory interventions for improving health and wellbeing.

## References

[B1] Garber C. E. Blissmer B. Deschenes M. R. Franklin B. A. Lamonte M. J. Lee I.-M. . (2011). Quantity and quality of exercise for developing and maintaining cardiorespiratory, musculoskeletal, and neuromotor fitness in apparently healthy adults: Guidance for prescribing exercise. Med. Sci. Sports Exerc. 43, 1334–1359. doi: 10.1249/MSS.0b013e318213fefb 21694556

[B2] Illidi C. R. Romer L. M. Johnson M. A. Williams N. C. Rossiter H. B. Casaburi R. . (2023). Distinguishing science from pseudoscience in commercial respiratory interventions: An evidence-based guide for health and exercise professionals. Eur. J. Appl. Physiol. 123, 1599–1625. doi: 10.1007/s00421-023-05166-8 36917254 PMC10013266

[B3] Sarnak M. J. Levey A. S. Schoolwerth A. C. Coresh J. Culleton B. Hamm L. L. . (2003). Kidney disease as a risk factor for development of cardiovascular disease: A statement from the American Heart Association Councils on Kidney in Cardiovascular Disease, High Blood Pressure Research, Clinical Cardiology, and Epidemiology and Prevention. Circulation 108, 2154–2169. doi: 10.1161/01.CIR.0000095676.90936.80 14581387

[B4] Vranish J. R. Bailey E. F. (2015). Daily respiratory training with large intrathoracic pressures, but not large lung volumes, lowers blood pressure in normotensive adults. Respir. Physiol. Neurobiol. 216, 63–69. doi: 10.1016/j.resp.2015.06.002 26112283

[B5] Wojan F. Stray-Gundersen S. Nagel M. J. Lalande S. (2021). Short exposure to intermittent hypoxia increases erythropoietin levels in healthy individuals. J. Appl. Physiol. 130, 1955–1960. doi: 10.1152/japplphysiol.00941.2020 33955265

